# CORESS Feedback: Cases from the Confidential Reporting System for Surgery

**DOI:** 10.1308/rcsann.2025.0117

**Published:** 2026-01-01

**Authors:** HJ Corbett, W Hawkins

**Affiliations:** on behalf of the CORESS Advisory Board

## Abstract

CORESS is an independent charity. Past CORESS cases can now be identified using a searchable CORESS case database, available via our website (coress.org.uk). We are grateful to those who have provided the material for these vignettes, some of which come from NHS England and Getting It Right First Time reports. The online reporting form is available on the CORESS website, which also includes previous Feedback reports. Published cases are acknowledged by a Certificate of Contribution, which may be included in the contributor’s record of continuing professional development, or which may form part of appraisal or annual review of competence progression portfolio documentation. Contributions from surgeons in training are particularly welcome.

## Unfamiliar equipment leading to laparoscopic port injury

### Case 323

A 28-year-old woman who had had no prior abdominal surgery presented with recurrent lower abdominal pain and requested diagnostic laparoscopy when investigation options were discussed. On the day of surgery, the surgeon requested a particular 12 mm disposable port but the operating theatre staff were unable to find the requested port. Three alternative ports were shown to the surgeon, who selected the port they wanted to use.

An open technique was employed to insert the primary port at the umbilicus but the fascial opening was not quite wide enough and insertion required a ‘push’, which was followed by a ‘give’. Two further ports were inserted under vision. During inspection of the small bowel, small bowel content was seen and a bowel injury then identified. The umbilical port site was enlarged and the bowel injury repaired. Subsequent inspection of the original port and trocar revealed that it was not the port that the surgeon had intended to use, and that the port used had a cutting blade within it, which became exposed when pressure was applied to the port.

### Reporter’s comments

Thankfully, although the patient needed a prolonged stay in hospital, she made an uneventful recovery. During debrief, the surgeon noted that they were not familiar with the port they had used and did not realise that there was a cutting blade within it. The theatre staff were sure that they had opened the port that was requested. The lesson here is in checking the equipment thoroughly, and even though the ports were discussed during the initial team brief/huddle and mentioned at ‘time out’, the correct port was not opened.

### CORESS comments

This was a ‘near miss’ in that a more catastrophic injury to the vasculature could have occurred. Modern-day surgery has seen a huge increase in disposable equipment, with many of these pieces of equipment having their own particular design features. Individual surgeon preferences, combined with supply chain issues, increase the likelihood of the surgical team encountering unfamiliar equipment. The ‘team brief’ step of the revised National Safety Standards for Invasive Procedures (NatSSIPs2) sequential standards is the appropriate time to discuss (and if possible inspect) the available equipment.^1^ Consideration should be given to postponing the case if suitable equipment is not available or if lack of training will put patient safety at risk.

**Figure:**
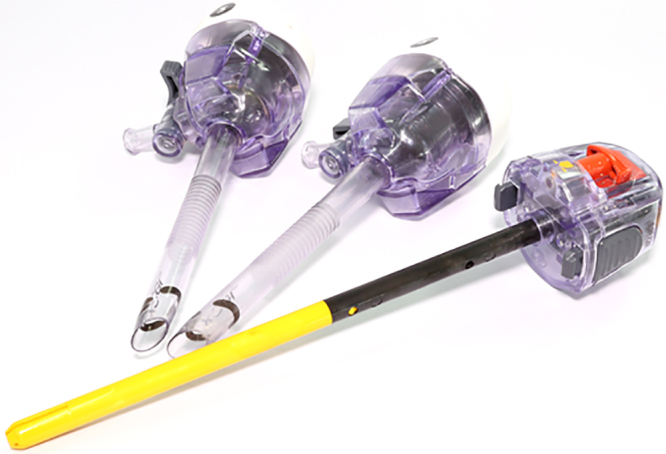
Image credit: SARASH SOOM-IM/Shutterstock.com

Reference1.Centre for Perioperative Care. The National Safety Standards for Invasive Procedures (NatSSIPs). https://cpoc.org.uk/guidelines-resources-guidelines/national-safety-standards-invasive-procedures-natssips
(cited November 2025).

## Wrong-side varicose vein treatment

### Case 324

A 54-year-old woman was referred to the vascular clinic for symptomatic varicose veins. She was noted to have superficial incompetence with haemosiderin pigmentation and prominent varicosities in both legs although her discomfort affected principally the left leg. Local funding was agreed to treat the veins in her left leg and she was placed on a pooled list for radiofrequency ablation.

The patient subsequently attended for day-case radiofrequency ablation, where consent for surgery for treatment of the left leg was obtained by the surgeon in training, who had read her outpatient notes. Veins in both legs were marked and the patient was taken to the operating theatre, where the veins of the right leg were treated by the consultant surgeon, who had not seen the patient previously. At postoperative follow-up, the patient queried why the left-sided varicosities, which were the cause of her major symptoms, had not been treated.

### Reporter’s comments

This was a case of wrong-side surgery. Factors contributing to this included the fact that the patient had similar varicosities in both legs and both legs had been marked. The operating surgeon had not seen the patient before or reviewed the full medical records and did not confirm the side with the patient although a World Health Organization surgical safety check was carried out. Despite the fact that the procedure was carried out under tumescent local anaesthesia, the patient did not query the side of the procedure when the leg was being prepared for intervention.

### CORESS comments

It is not clear why both legs were marked when surgery was only planned for the left side, nor whether the person obtaining consent was present at the team brief. Diligent execution of the ‘site marking’, ‘team brief’ and ‘sign in’ steps of the revised National Safety Standards for Invasive Procedures (NatSSIPs2) sequential standards could have alerted the team to discrepancies between the consent, site marking and the patient’s understanding in this case, where a number of opportunities to correct errors were missed.^1^

Reference1.Centre for Perioperative Care. The National Safety Standards for Invasive Procedures (NatSSIPs). https://cpoc.org.uk/guidelines-resources-guidelines/national-safety-standards-invasive-procedures-natssips
(cited November 2025).

## Unexpected surgical step leading to retained specimen

### Case 326

The patient underwent laparoscopic high anterior resection for a sigmoid tumour on an elective operating list. The initial resection was performed without incident. However, on performing the anastomosis, it became necessary to resect an additional portion of the rectum to achieve a satisfactory end-to-end anastomosis and remove a rectal diverticulum. The additional 8 cm portion of resected rectum was parked to the side for removal after completing the anastomosis. The second resected specimen was not removed as planned and was left inside the abdomen at the end of the procedure. The operating surgeon realised this while driving home. The patient returned to theatre later that night for the specimen to be removed.

No harm came to the patient other than requiring further anaesthesia. Nevertheless, the specimen would have caused a significant problem if left in for several days. All of the World Health Organization surgical safety checklists and forms were completed appropriately, and the trust’s duty of candour policy was employed.

### Reporter’s comments

The issue of failure to remove the second specimen only came to light when the surgeon remembered the fact as they drove home. This case might highlight the development of checklist fatigue or the inability for a checklist to cover every scenario without being unduly long. Alternatively, it may just have been an oversight that could not have been mitigated by the current checklist system. It was a very long operating day and the opportunities for breaks were limited; both the surgeon and the surgical team were tired. The case was discussed at the departmental morbidity and mortality meeting but no firm solution to prevent a recurrence was identified.

### CORESS comments

The revised National Safety Standards for Invasive Procedures (NatSSIPs2) sequential standards include a ‘reconciliation’ step (commonly called the ‘surgical count’), when swabs, sharps and instruments are accounted for at the end of procedure.^1^ The standard states that the count “should include any item that enters the procedural field” and the ‘sign out’ stage includes a step to check that specimens are labelled correctly. However, neither of these parts of the checklists are designed to detect what was an unexpected step in the surgical procedure, which resulted in an additional organic ‘item’. The extended surgical day was a confounder given that the team were tired and even a more extensive ‘sign out’ checklist may not have prevented the incident. Vigilance is key. Noting additional steps during the procedure on the theatre whiteboard is recommended to increase awareness among the entire theatre team.

Reference1.Centre for Perioperative Care. The National Safety Standards for Invasive Procedures (NatSSIPs). https://cpoc.org.uk/guidelines-resources-guidelines/national-safety-standards-invasive-procedures-natssips
(cited November 2025).

## Delayed diagnosis of testicular torsion

### Case 327

A 16-year-old young man complained of testicular pain at around 9.30pm but went to bed as usual as the pain was not severe. He was woken at 2am by severe pain and his mother drove him to the local emergency department. He vomited several times on the way. Unfortunately, the emergency department to which he presented did not have surgical services on site so an emergency transfer was arranged as he was suspected to have testicular torsion.

The young man arrived at the tertiary hospital at 6am and was assessed by the on-call surgeon in training at 6.30am. The pain had eased somewhat and a diagnosis of epididymitis was made as the epididymal tissue was very swollen. The surgeon in training recommended ultrasonography “in hours” and at handover, they told the day team of the plan. The ultrasonography was performed at 11.30am and although testicular torsion was identified, the surgical team were not notified. The on-call consultant reviewed the patient at 1.30pm and arranged emergency scrotal exploration within 30 minutes. The testis was torted and of doubtful viability but retained as there was some bleeding when the capsule was incised. However, atrophy followed over the next 3–4 months and the patient subsequently requested insertion of a testicular prosthesis.

### Reporter’s comments

The surgeon in training had already undertaken a scrotal exploration for torsion that night and during the subsequent case review, they noted that that influenced their decision making as they thought it was so unlikely to come across “two in a night”. The patient was not discussed with the on-call consultant at presentation. Morning handover was very busy and handover was inadequate: the patient’s history (high risk in view of age, severity of pain and vomiting) was not discussed, nor was the working diagnosis questioned, both of which were missed opportunities. There were further missed opportunities as the post-take ward round was delayed owing to another emergency and the radiologist did not flag the abnormal imaging. The patient made a successful claim.

### CORESS comments

This young man presented at a typical age with a history of severe testicular pain and vomiting. These red flag symptoms made testicular torsion highly likely. Prompt exploration was required and could have been achieved within the ideal six-hour window (assuming significant interruption of blood flow at 2am) despite the interhospital transfer. Although some units would request urgent out-of-hours preoperative ultrasonography, in a case like this, the history was sufficiently concerning that immediate exploration to detort the testis was the right course of action. Even urgent ultrasonography (to diagnose torsion) would almost certainly cause undue delay so this is not recommended with red flag symptoms. If in doubt, discussion with the on-call consultant is advised, whatever the time of day. Adequate handover is critical.

## Spinal drain issues

### Case 328

*This case was reported to the National Reporting and Learning System, and has subsequently been discussed by the CORESS Advisory Board.*

Ischaemic spinal cord injury is a significant complication of thoracoabdominal aortic surgery, especially in older patients, those with comorbidities or previous aortic surgery and those requiring emergency surgery. Neurological deficit usually presents immediately after surgery. Lumbar cerebrospinal fluid (CSF) drainage with a spinal drain has been shown to reduce the risk of ischaemic spinal cord injury following aortic surgery by optimising spinal cord perfusion.

A patient developed paraplegia after emergency aortic aneurysm repair. The spinal drainage was 0–3 ml per hour and the pressure 1–2 cm of water for some hours prior to the problem becoming evident. During spinal drain safety checks at 10pm, it was noticed that a filter was connected between the spinal drain tubing and a three-way tap. This filter should have been connected to the injection port for intrathecal medication, not within the drainage tubing. After correcting the position of the filter, the spinal drain pressure increased to 35 cm of water and drainage was 10 ml in 15 minutes. Total drainage from 8am to 10pm (before the filter was moved) was just 17 ml. Following removal of the filter, drainage was 47 ml in 3 hours. Lower limb sensation improved but limb movement did not recover.

### CORESS comments

Spinal drains may be sited for different reasons. Drains sited for the purpose of giving medication (such as intrathecal chemotherapy) should have a filter through which the medication passes before reaching the CSF. However, when sited following aortic surgery, the purpose is to drain CSF, which a filter will impede. Education regarding the critical difference in the two indications for a lumbar drain is vital. Considering the natural turnover of critical care staff, there should be regular training sessions that familiarise staff with all aspects of spinal drain indications and management. The World Federation of Societies of Anaesthesiologists has an online tutorial that is a valuable educational resource.^1^

**Figure:**
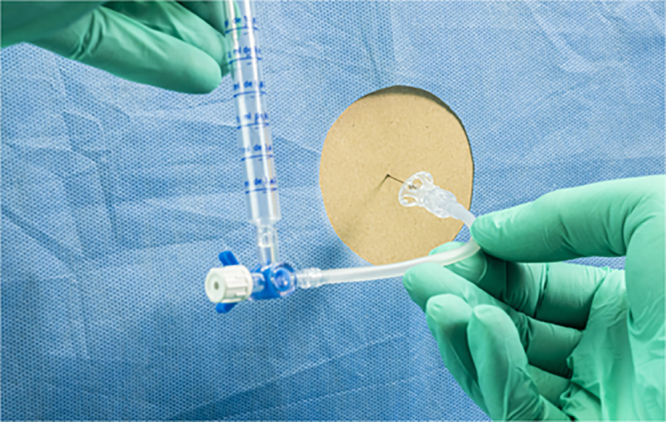
Image credit: DIGICOMPHOTO/Science Photo Library via Getty Images

Reference1.World Federation of Societies of Anaesthesiologists. Lumbar spinal drains for thoracoabdominal aortic surgery. https://resources.wfsahq.org/atotw/lumbar-spinal-drains-for-thoracoabdominal-aortic-surgery
(cited November 2025).
